# Decadal abundance patterns in an isolated urban reptile assemblage: Monitoring under a changing climate

**DOI:** 10.1002/ece3.9081

**Published:** 2022-07-05

**Authors:** Richard A. How, Mark A. Cowan, Jason R. How

**Affiliations:** ^1^ School of Human Sciences The University of Western Australia Perth Western Australia Australia; ^2^ Department of Terrestrial Zoology Western Australian Museum Perth Western Australia Australia; ^3^ Department of Biodiversity, Conservation and Attractions Perth Western Australia Australia; ^4^ Department of Primary Industries and Regional Development Perth Western Australia Australia

**Keywords:** assemblages, decades, extinctions, isolate, populations, rainfall residuals, relative abundance, reptiles, richness

## Abstract

Determine seasonal, annual, and decadal patterns of abundance in reptile species and assemblages occupying central Bold Park (~338 ha), an isolated urban bushland remnant in Perth, Southwestern Australia. Fenced pitfall trapping in four sampling sites, representing different habitats and fire history, over the primary reptile activity period for 35 consecutive years with over 17,000 individuals captured during 3300 days of sampling; the trapping regime was modified for the last 28 years. Sampling occurred in one of 35 global biodiversity hotspots that has a Mediterranean climate experiencing a 15% decline from the century average rainfall over the last 50 years. Twenty‐nine species were recorded, with 16 captured in 32 or more years and accounting for nearly 97% of all captures; the six most common for 81%. Three taxa became locally extinct. Activity predominates in warmer and dryer months (October to April), peaking in November–December. Species richness remained relatively constant between years with around 73% of known taxa captured annually. Assemblages did not change when analyzing the presence/absence data but moved through five statistically significant assemblages analyzing relative abundance data. Over the last 28 years, relative abundance was significantly and positively correlated with annual rainfall residuals, uniquely for the 4 years preceding annual sampling, resulting in significant changes in total assemblages and significantly similar patterns in four sample sites; the presence/absence data indicated only minor assemblage changes across sites. The number of species recorded annually remained relatively constant, but relative abundance illustrated significant temporal changes in assemblages over decades. The modeled relationship between relative abundance and annual rainfall residuals for 4 years preceding annual sampling is supported by known ecological responses and reptile demographics within this Mediterranean climate. Maintenance of urban biodiversity should consider impacts of a significantly drying climate exacerbating the extinction debt already inherent in isolated bushland populations experiencing limited immigration.

## Graphical abstract

Annual monitoring of an urban reptile assemblage over 35 years showed significant changes in relative abundance of individuals correlated with annual rainfall residuals for preceding years. Although local extinctions occurred, annual species numbers remained relatively constant throughout despite rainfall regime being around 15% below the 100 year mean.

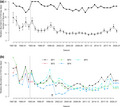

## 1 INTRODUCTION

European occupation of Australia's west coast in 1829 commenced a period of major modification to natural landscapes through intensive farming, introduction of exotic species, urbanization, and greatly altered fire regimes (Jarvis, 1986). These novel anthropogenic modifications were imposed on environments and ecosystems that had evolved during the Pleistocene glaciation cycles. Over the last 50 years, a significant 15% decline in mean annual rainfall (Bureau of Meteorology, 2020), now falling more evenly throughout the year (White et al., 2021), has further impacted these changed environments and their biota. Southwestern Australia is also a defined global biodiversity hotspot (Myers et al., 2000) experiencing a Mediterranean climate with cool wet winters and hot and dry summers. Within this hotspot, the city of Perth extends across the Swan Coastal Plain and covers all coastal landforms and the adjacent Darling Range.

It is axiomatic that human activities fundamentally alter biodiversity, but it is how biodiversity changes over time that provides the best guidance for implementing conservation measures and distinguishing change that can be attributed to external factors from underlying natural variability (Gotelli et al., 2017; Magurran et al., 2010). Marked anthropogenic impacts modify species abundances and selection pressures operating on local populations and ecosystems, especially in rapidly expanding urban areas (Alberti, 2015; Blowes et al., 2019). A survey of the biota of the northern Swan Coastal Plain in 1978 showed 45 percent of the natural vegetation, 37 percent of native mammal species, and several indigenous birds had been lost in 150 years since European occupation (Western Australian Museum, 1978). Subsequent studies within metropolitan Perth determined that over 75% of vegetation had been cleared, 73% of native mammal species had become locally extinct, and 44% of bird, 33% of reptile, and 27% of amphibian species had declined in distribution (How & Dell, 2000; Stenhouse, 2004).

MacArthur and Wilson (1967) highlighted the relationship between species richness and island area, factors that are now inherent in fragmented mainland natural landscapes associated with rapid expansion of agricultural and urban areas. They also indicated that immigration was a fundamental aspect of island equilibrium and this interacted with island size and distance to source. Debate continues, however, in the literature as to whether habitat loss or fragmentation per se are the major drivers of species loss (Matthews et al., 2014) and what role connectivity between fragments plays in maintaining the evolutionary processes essential to the persistence of populations and assemblages in isolated natural habitats (Carroll et al., 2015). What is less clearly documented is the temporal sequence of change to species richness and abundance and how time impacts the gradual changes to biotic assemblages (Magurran et al., 2019; Ramalho & Hobbs, 2012); changes that are fundamental to distinguishing variation attributable to external factors, such as climatic or anthropogenic induced modifications, from underlying natural change (Gotelli et al., 2017; Magurran et al., 2010).

Ectotherms, such as reptiles, have poor dispersal abilities and make good models for assessing how environmental changes impact populations and assemblages in different communities (Ackley et al., 2015; Doherty et al., 2020; Driscoll, 2004; How & Dell, 1994; Jellinek et al., 2004; Kitchener et al., 1980). Native vegetation reserves in the Western Australian Wheatbelt region had a strong positive correlation between reptile species richness and reserve area (Kitchener et al., 1980). This finding contrasts with overall results from a global review of reptiles and environmental variables (Doherty et al., 2020) that determined modification to habitats was the principal cause of declines in populations. On the Swan Coastal Plain, snake and total lizard richness was significantly correlated with fragment area, although skinks alone were not, suggesting that over the last 170 years of anthropogenic modification, taxon‐specific local extinctions have occurred in smaller natural bushland remnants (How & Dell, 2000). Again, most studies focus on the spatial relationships between reptile richness and abundance while studies of temporal change in assemblages remain more limited (Dornelas et al., 2014).

Bold Park is the largest and most biodiverse inner urban bushland remnant of Perth (How & Dell, 1990, 1994) and lies central in both global (Southwestern Australia) and regional (Swan Coastal Plain) biodiversity hotspots that are experiencing a significantly drying climate. An initial study of Bold Park (How, 1998) documented the richness and activity of reptile species within different habitats over monthly and seasonal timeframes as a corollary to a broader regional survey of isolated native bushlands (How & Dell, 2000). The continuation of the Bold Park study provides a unique opportunity to appraise long‐term temporal changes in reptile species populations and assemblages and evaluate those changes in a bushland reserve that has remained unchanged in size and management modifications for many decades, but has experienced altering climatic conditions, particularly decreasing rainfall.

## 2 METHODS

### 2.1 Sample location and sites

Bold Park encompasses 437 ha of coastal bushland within the Perth Metropolitan Area of Western Australia [31^o^56'40”S/115^o^46'17″E]. The Park has been gazetted by the Western Australian Government as an A Class Reserve for over 60 years, being protected and managed for passive (pedestrian) recreation and biodiversity conservation by the Perth City Council and more recently by the Botanic Gardens and Parks Authority. The Park is enclosed on three sides by urban development, sporting fields, public open space, and golf courses, while in the west it abuts the Indian Ocean. The contiguous central section of 338 ha is bounded by dual‐lane bitumen roads and is traversed by numerous packed‐earth tracks for pedestrian use only. The Park supports a diverse array of native vegetation comprising nine plant communities and a rich flora of 232 non‐native and 310 native plant species on principally calcareous sandy soils; Tuart and Banksia woodlands, as well as heaths on limestone, predominate the nine plant communities (Keighery et al., 1990).

Four sites were selected for sampling in 1986 that comprised different principal vegetation formations, covering the greatest variability of the Park's heterogeneity. Sites represented mixed coastal heath (BP1), *Dryandra sessilis* shrubland (BP2), *Banksia attenuata/B. menziesii* low woodland (BP3), and *Eucalyptus gomphocephala* (Tuart) woodland (BP4). *Dryandra* shrubland (BP2) was substituted in 1993 by mixed inland heath (BP5) to expand the geographic coverage and diversity of soil and habitats sampled.

Dunstan (2013) appraised historical panchromatic photographs from 1963 of Bold Park and documented an absence of fires on the selected sampling sites since before that time, with the exception of the intense fire that burnt all of the Tuart Woodland (BP4) site and nearly 100 hectares adjacent in December 2000.

### 2.2 Monitoring regime and duration

**FIGURE 1:**
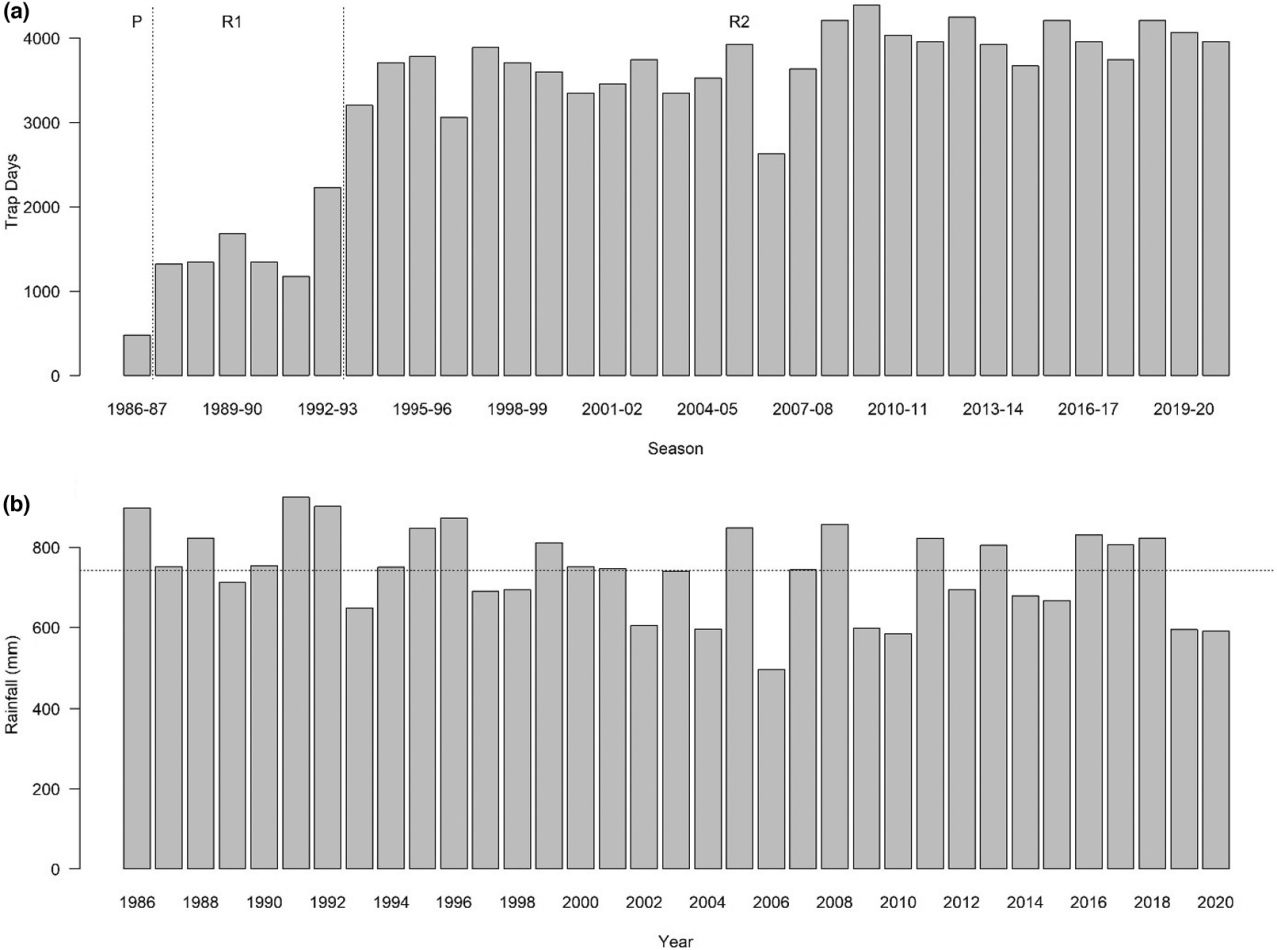
(a) Number of trap days per sampling period, with the demarcation between the pilot (P) and two sampling regimes (R1 & R2) denoted by vertical dotted lines. (b) The annual recorded rainfall and the mean (horizontal line) during the study period

Fenced pitfall trapping was used throughout the study, predicated on its known success in capturing reptile species in the predominant sandy substrates of the Swan Coastal Plain (How & Dell, 2000). Pilot sampling occurred in 1986–1987, involving four consecutive days of trapping in 5 months—December 1986, February, March, May, and June 1987 at four sites (Pilot: How, 1998). Sites comprised six pitfall traps of PVC pipes (15 cm diameter and 60 cm depth) spaced eight meters apart under a 50‐m‐long 30‐cm‐high fly‐wire fence, which bisected each trap. Temporal sampling was refined and expanded in September 1987 (Regime 1; Figure 1a) to encompass a period covering over 50 days within 8 months of the year (September–April). This austral spring–autumn sampling period (described as overlapping years) covered the next six consecutive years until 1992–1993.

In September 1993, the type (20 liter buckets 29 cm diameter 39 cm depth) and configuration of pitfall traps (nine traps spaced 10 m apart and arranged in a 3 × 3 grid) at each site was changed (Regime 2; Figure 1a). Each trap was overlain by a 7‐m‐long 30‐cm‐high fly‐wire fence. Site BP2 was replaced at the commencement of this regime with BP5. The regime change was necessitated to standardize methodology with the broader study of the ground fauna across the Perth metropolitan region between 1993 and 1997 (How & Dell, 2000) and involved an increase in effort from around 1500 trap days to over 3000 trap days during subsequent years (Figure 1a). Biological data were collected by RAH across both sampling regimes and involved individual identification (to species level) and recording sex and selected body measures before release within two meters of the pitfall trap.

### 2.3 Analyses

Analysis of assemblage data was conducted using the Primer E software package (Clarke & Gorley, 2006). Assemblage relationships between years were analyzed using species presence/absence data and the more ecologically informative relative abundance data collected. Presence/absence data were examined using the Sorenson's Index of similarity with UPGMA clustering. Relative abundance (square‐root transformed) data were calculated as species captures per 100 pit‐trap days for every sampling year and site to account for variation in sampling effort between years. Assemblage comparisons used the zero‐adjusted Bray–Curtis dissimilarity index and UPGMA clustering (Clarke et al., 2006). The *Simprof* routine determined significantly different assemblages, which is driven by a shift in the species composition or their relative abundance between sampling events. The *RELATE* routine examined the similarity between the nMDS assemblage matrices of different sites.

Relative abundance (*n* per 100 trap days) was determined for all reptiles for each month sampled in each year and is a proxy for absolute abundance. As there are differences in activity between months (Figure 4) and variation in the amount of sampling in each month between years, it was necessary to generate an index which was standardized to account for variation in sampling effort. The relative abundance was log transformed and standardized using a generalized linear model (GLM) with factors (*f*) of sampling period (*f*; from 1993–1994 to 2020–2021) and month (*f*; September to April) as per the equation below (Equation 1).

(1) $$\log_{e}\left(U\right)=\sum_{j=1}^{p}x_{j}\beta_{j}+\varepsilon$$

where *U* is capture rate (*n*/100 trap days), $x_{j}$ are the *p* explanatory variables including quantitative and qualitative variables, $\beta_{j}$ are estimated coefficients, and $\epsilon \sim N\left(0,\sigma^{2}\right)$ is the error term.

Marginal means were extracted for the factor being examined, with data presented as back‐transformed capture rates. Analysis was undertaken using R (R Core Team, 2020) with marginal means extracted following (Lenth, 2021). Relative reptile abundance was compared with annual rainfall residuals for 6 years proceeding sampling years (Y0 to Y6), using a GLM, where Y0 is the residual for the year immediately preceding sampling, Y‐1 the year prior, etc. Model simplification occurred through a forward and backwards stepwise algorithm assessing models on the AIC.

## 3 RESULTS

### 3.1 Rainfall pattern of Perth and Bold Park

Rainfall data were collated from weather stations ~2 km from Bold Park, Subiaco Treatment Plant (pre‐1993) and Swanbourne (1993 onwards). The long‐term records (1876–1992) were from Perth Regional Office, approximately 8 km east of Bold Park, and indicated an annual mean of 873 mm. The mean annual rainfall over the study period at Bold Park (742 mm) represents only 85% of the long‐term mean for Perth (Figure 1b).

### 3.2 Species

Twenty‐nine reptile species representing eight families were captured between 1986 and 2020 in Bold Park (Table 1). Over 35 years 17,485 reptile individuals were captured over 3317 days of trapping (94.77 ± 23.4 days annually) comprising 114,624 pit‐trap days (3275 ± 1053 pit‐days annually).

**TABLE 1:** Total number of individuals of reptile species captured over 35 years sampling in Bold Park and the numbers captured in discrete monitoring sites. The number of trapping days and pitfall trap days undertaken are also documented. The bracketed number in each column is the number of years each taxa was recorded in that site

	BP1	BP2	BP3	BP4	BP5	TOTAL
Trapping Days	3317	399	3317	3317	2918	3317
Pit‐days	28,656	2394	28,656	28,656	26,262	114,624
**Elapidae**						
*Echiopsis curta*	2 (1)	– (0)	– (0)	– (0)	– (0)	2 (1)
*Pseudonaja affinis*	11 (7)	1 (1)	9 (9)	2 (2)	8 (6)	31 (18)
*Simoselaps bertholdi*	192 (35)	24 (7)	282 (35)	132 (30)	180 (28)	810 (35)
*Neelaps bimaculatus*	35 (20)	1 (1)	34 (16)	30 (14)	44 (19)	144 (32)
*Neelaps calonotos*	18 (13)	4 (2)	28 (19)	10 (8)	13 (10)	73 (28)
*Brachyurophis fasciolata*	11 (6)	1 (1)	3 (2)	1 (1)	2 (2)	18 (8)
*Brachyurophis semifasciata*	85 (30)	2 (1)	42 (18)	19 (14)	49 (22)	197 (35)
**Typhlopidae**						
*Anilios australis*	110 (29)	11 (6)	148 (33)	77 (25)	63 (23)	409 (34)
**Agamidae**						
*Pogona minor*	179 (34)	2 (2)	136 (30)	11 (6)	35 (15)	363 (34)
*Ctenophorus adelaidensis*	10 (8)	16 (4)	21 (9)	1 (1)	53 (6)	101 (16)
**Diplodactylidae**						
*Lucasium alboguttatum*	1 (1)	– (0)	– (0)	– (0)	5 (3)	6 (4)
*Strophurus spinigerus*	1158 (35)	35 (6)	445 (35)	15 (12)	1170 (28)	2823 (35)
**Gekkonidae**						
*Christinus marmoratus*	3 (3)	– (0)	14 (10)	27 (12)	5 (5)	49 (21)
*Heteronotia binoei*	– (0)	– (0)	10 (6)	– (0)	– (0)	10 (6)
**Pygopodidae**						
*Aprasia repens*	27 (15)	2 (2)	1 (1)	144 (32)	3 (3)	177 (34)
*Lialis burtonis*	58 (25)	24 (7)	105 (31)	44 (22)	60 (23)	291 (35)
*Pletholax gracilis*	2 (2)	– (0)	– (0)	– (0)	1 (1)	3 (3)
**Scincidae**						
*Cryptoblepharus buchananii*	29 (13)	4 (4)	102 (29)	164 (33)	9 (7)	308 (35)
*Ctenotus fallens*	1383 (35)	81 (7)	792 (35)	359 (35)	793 (28)	3408 (35)
*Ctenotus australis*	60 (15)	3 (2)	22 (12)	7 (5)	37 (12)	129 (23)
*Cyclodomorphus celatus*	41 (19)	– (0)	8 (5)	5 (5)	15 (11)	69 (29)
*Hemiergis quadrilineata*	589 (35)	267 (7)	1166 (35)	1087 (35)	747 (28)	3856 (35)
*Lerista elegans*	315 (34)	40 (7)	439 (34)	567 (35)	397 (28)	1758 (35)
*Lerista lineopunctulata*	547 (35)	38 (7)	363 (35)	251 (33)	297 (28)	1496 (35)
*Lerista praepedita*	114 (29)	6 (3)	88 (30)	5 (5)	67 (24)	280 (34)
*Menetia greyii*	21 (16)	5 (4)	55 (18)	323 (35)	13 (11)	402 (34)
*Morethia obscura*	– (0)	– (0)	7 (5)	3 (3)	1 (1)	11 (8)
*Tiliqua rugosa*	66 (28)	15 (6)	29 (18)	41 (22)	50 (21)	201 (35)
**Varanidae**						
*Varanus gouldii*	19 (12)	1 (1)	11 (7)	16 (10)	25 (17)	72 (27)
Total species	27	22	26	25	27	29
Total individuals (ind)	5096	583	4360	3341	4142	17,485
Ind/100 pit–days	17.783	24.353	15.215	11.659	15.778	15.254
Mean species annually	15.4 ± 2.7	13.0 ± 2.9	14.8 ± 2.1	12.4 ± 2.2	14.6 ± 2.4	21.3 ± 2.1
Percent of Total Species	57.0	59.1	56.9	49.6	54.1	73.3

The capture profile of species within the assemblage shows 15 species were captured during the pilot study year (1986–1987), 24 species by the end of the second year, and 26 by end of the fourth year. The 27th and 28th species were first captured during the eighth and 13th year, respectively. The 29th species, a gecko, *Heteronotia binoei*, first captured in the 30th year after 2744 days of sampling, has been recaptured each subsequent year suggesting a recent colonization by this indigenous, widespread, and parthenogenic taxon. In addition, several species have not been recorded for over two decades. The snake, *Echiopsis curta* was last captured 27 years ago (2845 trapping days), the gecko, *Lucasium alboguttatum* 25 years ago (2705 trapping days) and the snake, *Brachyurophis fasciolata* 21 years ago (2306 trapping days). The dragon, *Ctenophorus adelaidensis*, has been captured only once in the last 17 sampling years (2029 trapping days) and has probably disappeared from the assemblage. On 10 occasions, five or more years elapsed between recaptures of a species.

The average annual species captured was 21.30 ± 2.1 (*n* = 35), defining that around 73% of the total assemblage was captured during any sampling year. Eleven species were captured in every one of the 35 years, a further four in 34 years, and one in 32 years. These 16 common and abundant taxa account for 96.9% of all reptile captures (Table 1) and exhibit the least variation in abundance between years. The six most frequently captured taxa—*Hemiergis quadrilineata* (22.05%)*, Ctenotus fallens* (19.49%), *Strophurus spinigerus* (16.15%)*, Lerista elegans* (10.05%)*, L. lineopunctulata* (8.56%), and *Simoselaps bertholdi* (4.63%)—comprised nearly 81% of all captures.

A further eight species were captured for between 16 and 29 years and showed greater annual variation in abundance. The remaining five species were captured in eight or fewer years and demonstrated the greatest variation annually. Less frequently captured species were either genuinely rare (e.g., *Pletholax gracilis* and *Lucasium alboguttatum*), unsuitable for capture using the methodology (e.g., *Pseudonaja affinis*) or able to climb from traps (e.g., *Christinus marmoratus*).

### 3.3 Assemblages

**FIGURE 2:**
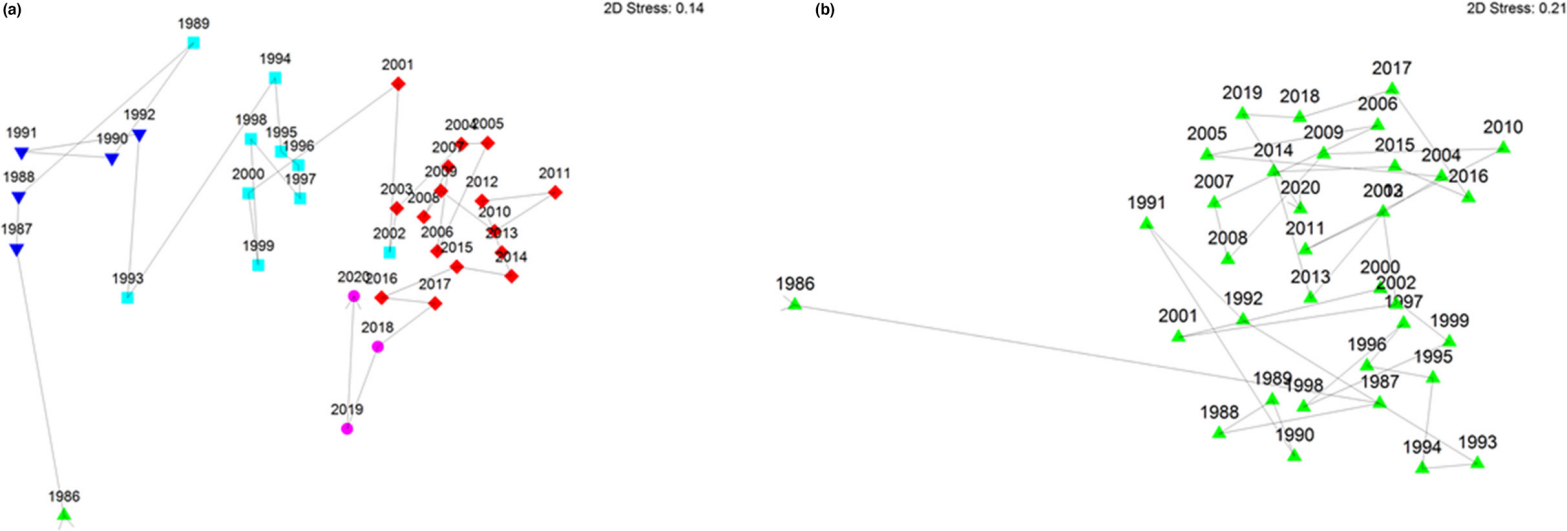
Yearly relationships of reptile assemblages in Bold Park from 1986–1987 to 2020–2021. (a) nMDS plot analyzing effort‐adjusted abundance with square root transformation data and a zero‐adjusted Bray–Curtis dissimilarity measure with UPGMA clustering; (b) nMDS plot analyzing species richness data using Sorensen similarity measure and UPMGA clustering. Symbols represent significantly different assemblages, and arrows represent temporal trajectory

Relative abundance data of reptiles in Bold Park indicated five significantly different assemblages occurred over the study. One assemblage was unique to the pilot study year (1986–1987), while the other four different assemblages followed in a broadly chronological sequence during the subsequent 34 years, showing slight overlap between the two trapping regimes (Figure 2a). Presence/absence data showed no significant change in the Park's reptile assemblage over the 35 years (Figure 2b).

#### 3.3.1 Assemblages on sampling sites

Assemblages on sampling sites were analyzed separately for the first 7 years (Pilot and Regime 1) and the last 28 years (Regime 2) in order not to confound potential changes resulting from altered trapping methodology.

##### Regime 1

**FIGURE 3:**
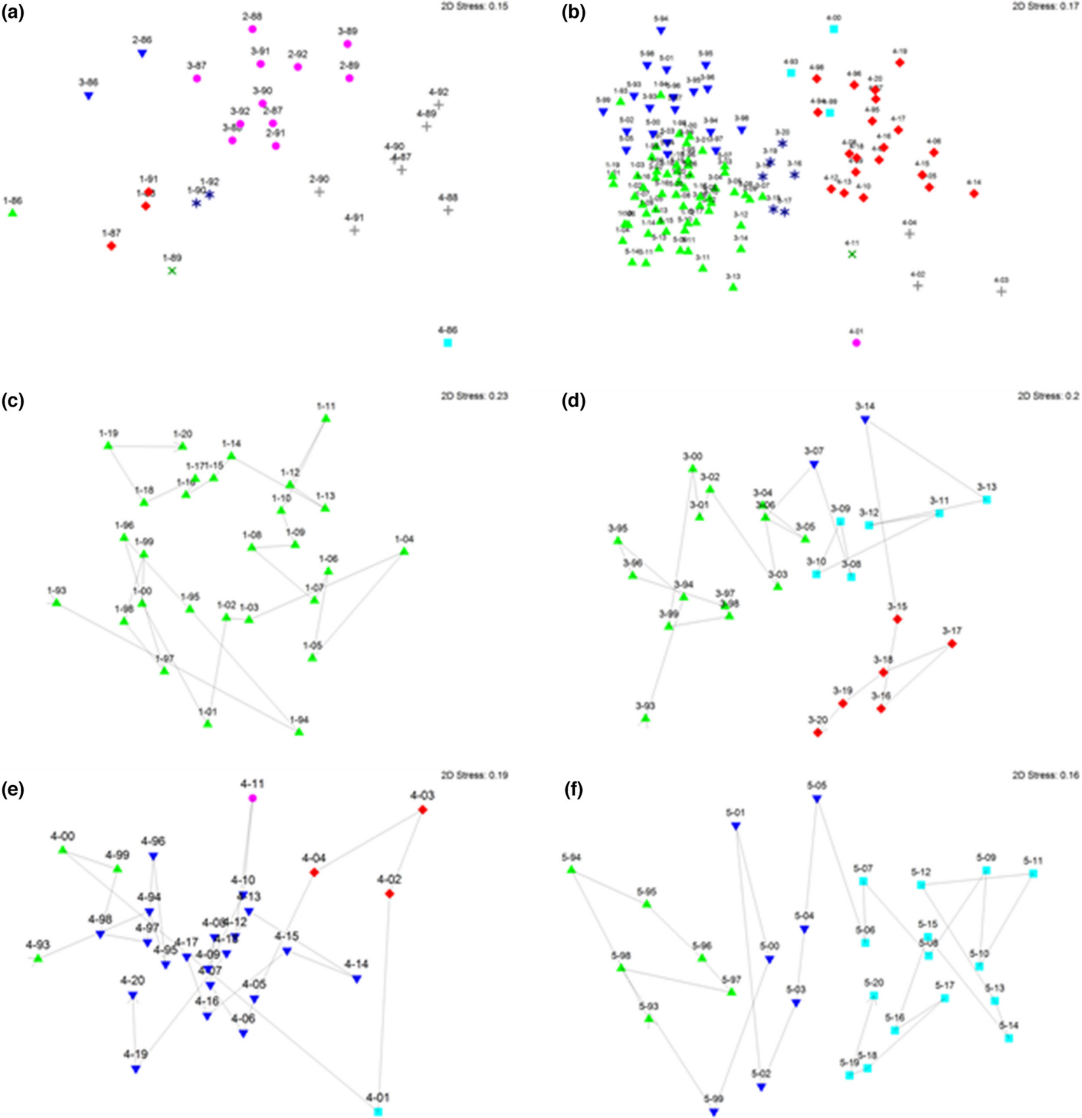
Reptile assemblage relationships for all sampling sites in Bold Park (a) from 1986–1987 to 1992–1993 and (b) from 1993–1994 to 2020–2021.Temporal change in assemblage relationships for individual sampling sites from 1993–1994 to 2020–2021 for BP1 (c), BP3 (d), BP4 (e), and BP5 (f) Plots are of nMDS analyzing effort‐adjusted abundance with square root transformation data and a zero‐adjusted Bray–Curtis dissimilarity measure with UPGMA clustering. Sites are identified by their number and year of sampling; different colored symbols represent significantly different assemblages; the directional line indicates sampling years in chronological sequence

Relative abundance defined eight statistically different site assemblages over the first 7 years of study (Figure 3a). Three assemblages were unique to the pilot study year—one each in BP4 and BP1 and the third shared by BP2 and BP3. The predominant Tuart Woodland (BP4) assemblage over the subsequent 6 years was shared only with *Dryandra* shrubland (BP2) in 1990–1991, while BP2 had a similar assemblage with Banksia Woodland (BP3) over five of the subsequent years. The near‐coastal heath (BP1) had three significantly different assemblages over the subsequent 6 years.

Presence/absence analysis documented just two significantly different assemblages, one unique to BP4.

##### Regime 2

Relative abundance indicated eight significantly different assemblages across sites (Figure 3b). Five were unique to BP4, and the other three were common to the remaining three monitoring sites. Three sites (BP1, BP3, and BP5) shared similar dominant and secondary assemblages (Figure 3c, d, f), contrasting with BP4 (Figure 3e) over those years. The BP1 assemblage did not differ significantly throughout the 28 years, while BP3 changed through four significantly different assemblages, BP5 through three, and BP4 through five assemblages.

Presence/absence analysis documented four significantly different assemblages; one unique to BP4 and another unique to all other sites; two assemblages were confined to specific sites during two separate years.

A significant correlation existed between the similarity matrices for all four sites (Table 2), and they showed similar patterns over time (Figure 3c–f), despite each site progressing through variable numbers of different assemblages over the 28 years.

**TABLE 2:** Rho (*ρ*) values and significance levels (*p*) from a comparison between the similarity matrices of four sampling sites between 1993–1994 and 2020–2021. Calculated from the RELATE program (Spearman correlation and 999 iterations)

Site	BP1‐93+	BP3‐93+	BP4‐93+
BP1‐93+			
BP3‐93+	*ρ* = 0.228, *p* = 0.4%		
BP4‐93+	*ρ* = 0.289, *p* = 0.52%	*ρ* = 0.225, *p* = 1.6%	
BP5‐93+	*ρ* = 0.445, *p* = 0.1%	*ρ* = 0.391, *p* = 0.1%	*ρ* = 0.230, *p* = 1.1%

### 3.4 Activity and relative abundance

The pilot study showed activity among total reptiles was greatest over the austral late spring–summer months, so subsequent annual sampling periods were focused over that time (How, 1998). The significantly lower sampling effort during 1986–1987 (Figure 1a) likely contributed to markedly lower species richness, relative abundance, and unique assemblages (Figure 3a) in the four sampling sites for that year, supporting an omission of the pilot study year from further analysis.

Analysis revealed significant month and year effects on relative abundance (*n*/100 trap days) of reptiles captured through the 34 years of Regime 1 & Regime 2. To examine seasonal differences in relative abundance and variability in monthly samples between years, marginal means (accounting for differences in monthly sampling) were generated.

#### 3.4.1 Sampling activity

**FIGURE 4:**
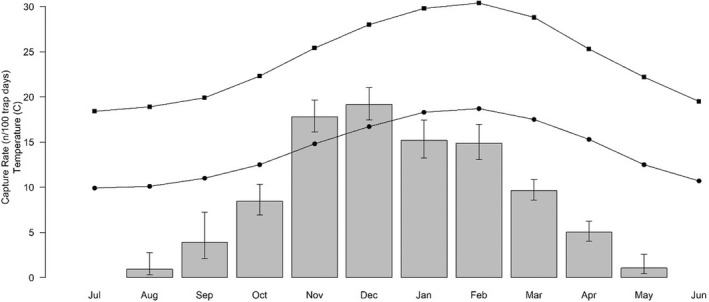
Marginal mean capture rate (*n*/100 trapdays ±95CI) for all months of sampling of reptiles at Bold Park (bar plot ±95%CI) and the mean monthly maximum (squares) and minimum (circles) temperature (°C) throughout the study period

Reptile capture rates across Bold Park peaked in November–December throughout the entire study, followed by a gradual decline through late summer and autumn months to a low through winter before increasing throughout spring. Peak activity preceded the February peak in seasonal temperatures (Figure 4).

#### 3.4.2 Relative abundance

**FIGURE 5:**
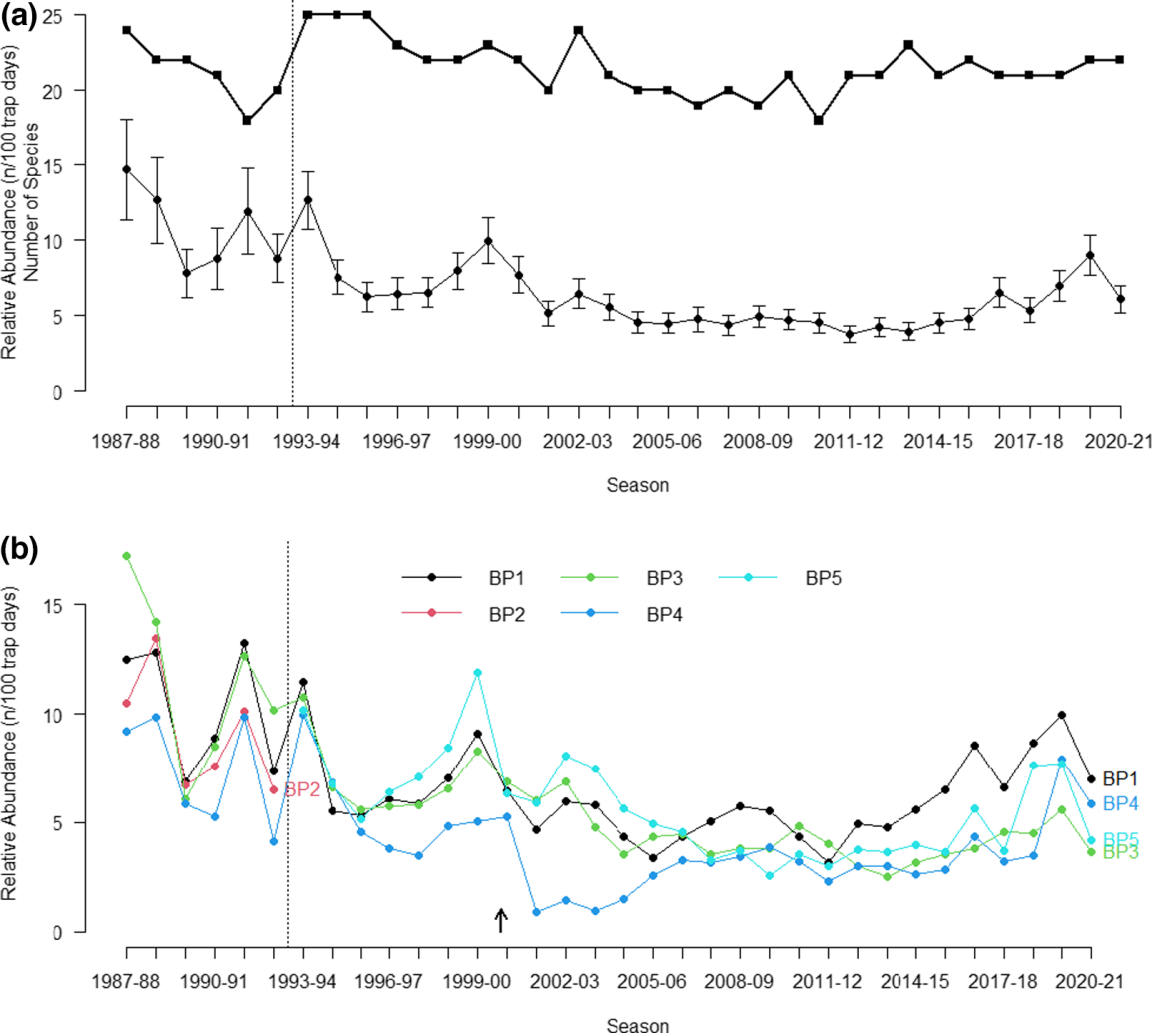
(a) Number of unique species recorded each year (squares, thick upper line) and overall relative abundance (*n*/100 trap days ±SE) for each annual sampling of reptiles at Bold Park (dots, thin lower line). (b) Relative abundance for each annual sampling at five sites at Bold Park. Dotted vertical line denotes the change in sampling regime and arrow the year fire burnt through BP4

The 6 years of sampling under Regime 1 (1987–1988 to 1992–1993) exhibited marked variation about annual estimates in relative abundance (Figure 5a). Following the changed trapping protocols (Regime 2) in 1993–1994, there was a marked reduction in the error associated with annual estimates and considerably less interannual variation. This component of the time series demonstrated a general pattern of decline in relative abundance that continued for around 25 years, reaching a nadir in 2011–2012. The ensuing 8 years showed a general increase in relative abundance, prior to the sharp decline in 2020–2021; the number of species captured, however, remained relatively stable over decadal cycles (Figure 5a).

The peaks in annual abundance within the entire assemblage were generally attributable to pronounced increases in the relative abundance of specific species. The increases during 1993–1994, 1999–1900, 2016–2017, and 2019–2020 were primarily a consequence of increased abundance of *Strophurus spinigerus* and, to a lesser extent, of *Ctenotus fallens* during 1999–2000, *Lerista lineopunctulata* during 1993–1994, and *L. elegans* during 2019–2020. Decreased abundance from the 1999–2000 peak during 2000–2001, and the following year, was the response in several species to the extensive fire in BP4 during December 2000, particularly a very marked decline in *Hemiergis quadrilineata*. This decline becomes obvious when relative abundance is examined in specific sites, notably in BP4 post‐fire (Figure 5b). Despite the 2000 fire's immediate impact, the pattern of change in overall relative abundance was generally similar between all sites over the entire study, irrespective of change trapping methodology (Figure 5b).

#### 3.4.3 Relative abundance and rainfall pattern

**FIGURE 6:**
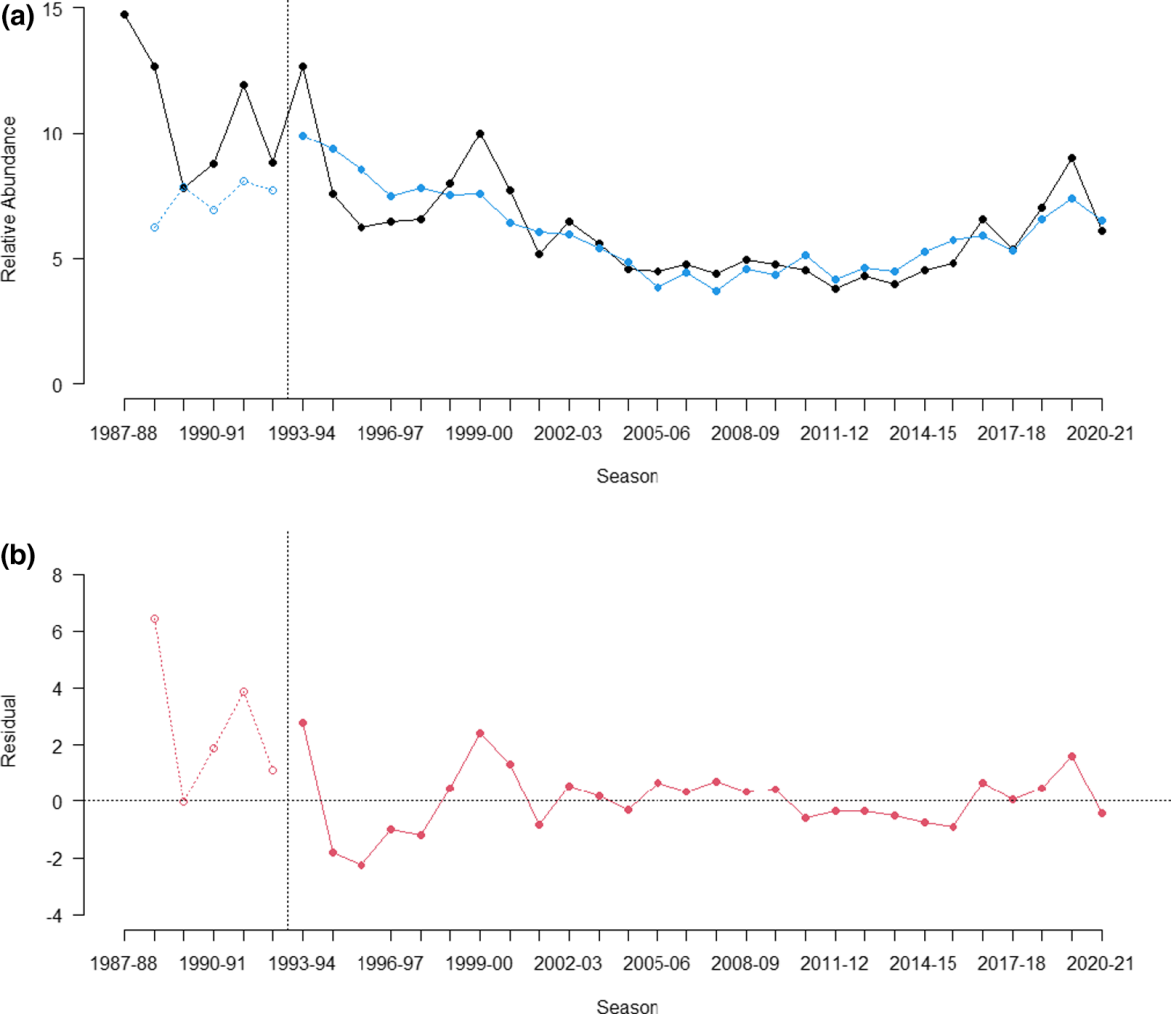
(a) Yearly relative abundance of reptiles (black) and the predicted abundance based off modeled rainfall (blue); (b) the residuals between the modeled and actual relative abundance. Dotted vertical line denotes change in sampling protocol, with open circles and dashed line where model coefficients were applied to sampling periods not included in the model

Significant positive relationships existed between rainfall residuals and reptile relative abundance for the 4 years preceding the sampling year. Due to the different sampling regimes (Regime 1) prior to the 1993–1994 period, and the greater variation in annual estimates and interannual variation (Figure 5a), modeling of rainfall was undertaken only on relative abundance from 1993–1994 onwards (Regime 2). The modeled estimated relative abundances from rainfall residuals closely aligned with relative abundance recorded in the pit traps over the last 28 years (Figure 6a). This 28‐year time series demonstrated that annual rainfall residual was highly significantly correlated with relative abundance (Table 3) for each of the four years prior to sampling (Years −1 to −4), but not the year in which sampling commenced (Year 0) or the years five and six prior to sampling (Year‐5 & ‐6). Notable deviations from the model occurred at the start of the time series (1993–1994 to 1995–1996), at the turn of the century (1999–2000 & 2000–2001) and in 2019–2020 (Figure 6b). Model estimates of relative abundance were also predicted for sampling Regime 1 (1987–1988 to 1992–1993) but resulted in lower estimates than the actual recorded relative abundance, resulting in considerable positive residuals (Figure 6b).

**TABLE 3:** Relationship between annual rainfall residual (from long‐term mean) and relative abundance over 28 sampling periods between 1993–1994 and 2020–2021 on Bold Park. The rainfall residual of the year when the sampling period commenced Yr(0) and preceding years Yr(–1 to –6) are numbered accordingly

Rainfall	Estimate	SE	*t* value	Pr(>|t|)
(Intercept)	1.776434	0.031961	55.582	<2e−16***
Residual Yr(0)	0.000256	0.00032	0.8	0.43328
Residual Yr(−1)	0.001282	0.00036	3.565	0.00194**
Residual Yr(−2)	0.001658	0.000341	4.869	9.29e−05***
Residual Yr(−3)	0.001871	0.000356	5.253	3.85e−05***
Residual Yr(−4)	0.000897	0.0003	2.992	0.00721**
Residual Yr(−5)	0.000476	0.000375	1.271	0.21847
Residual Yr(−6)	−0.00036	0.000326	−1.103	0.28329

## 4 DISCUSSION

Reptile species and populations are in decline in many ecosystems across the world (Doherty et al., 2020; Gibbons et al., 2000), principally due to anthropogenic induced perturbations. Habitat modification is a key factor in population declines though significantly different responses exist between species (Doherty et al., 2020). In urban areas, the extent of habitat fragmentation, infrastructure development, predation, and fire are principal threats to the persistence of native species and have a strong negative impact on both richness and diversity (Ackley et al., 2015; How & Dell, 1994; Jellinek et al., 2004). Habitat destruction, modification, and fragmentation are also the primary causes of decline in reptile richness and abundance in agricultural landscapes, with local extinctions frequent in both spatial and temporal datasets (Driscoll, 2004; Kitchener et al., 1980). An examination of 83 recent extinctions among reptiles (Slavenko et al., 2016) found that insularity was the main determining correlate (73 being island taxa) and that reptiles with larger body size were the most prone to extinction. Evaluation of extinction thresholds and extinction debt in meta‐populations (Hanski & Ovaskainen, 2002) determined that extinction of taxa is predicated by a lack of dispersal ability as well as habitat loss. These two factors also impact reptile populations and assemblages in fragmented native bushlands across the Swan Coastal Plain landforms (How & Dell, 1994). While both factors are key drivers, a deficit of long‐term studies has been unable, as yet, to examine shifts in climatic conditions on reptile communities. The strongly positive correlation between abundance and changes in annual precipitation over the 4 years preceding sampling indicates the likely long‐term responses of reptile assemblages to a changing climate. There has been a marked decline in rainfall over the last 50 years, specifically over last 35 years when it has been 15% below Perth's long‐term average. This decline reached 25% below the long‐term mean between 2006 and 2010, the driest five‐year period ever recorded (Bureau of Meteorology, 2020), indicative of a changing drying climate in the Perth region. Our results demonstrate that within a stable large bushland isolate, changing climate can have pronounced and explicable impacts on reptile abundance.

### 4.1 Methodological considerations

Annual capture rates of reptiles were interpreted to represent a simple relationship to relative abundance, given the extended consistency of sampling that provided comparable annual data for arboreal, epigeic, and fossorial taxa. However, relative abundance of reptiles (based on pitfall capture techniques) in similar Perth coastal environments differed from absolute abundance (determined from habitat destructive total counts) depending on species‐specific life history traits and habits (Bamford & Calver, 2015). To obviate the effects of changed trap type and intensity, our study evaluated assemblage structure separately for the first seven and latter 28 years of sampling. The first‐year pilot study illustrated sampling inadequacy for duration and intensity but provided crucial information for the establishment of future standardized protocols (How, 1998) and regional biodiversity surveys (How & Dell, 2000).

Annual species richness (α‐diversity) of reptiles in the Bold Park assemblage showed relative long‐term stability. Likewise, no significant changes occurred in assemblages when analyzing presence/absence data, despite changes in trap type and sampling intensity. However, relative abundance data documented five significantly different assemblages over the 35 years, including a unique assemblage in the pilot year.

Common species are focal in determining changes within assemblages because even relatively small declines in their abundance can result in proportionally large changes in overall individuals (along with biomass), which in turn disrupts assemblages and probably their ecological function in ecosystems (Gaston & Fuller, 2008). Gaston and Fuller also documented the value in focusing on numerical changes in common species because they play the more significant role in determining assemblage structure than rare species. Many assemblage studies have focused on species presence/absence that over‐represent taxa, which are either rare or infrequently sampled, while simultaneously under‐representing common species and their functional roles in the ecosystems. Comparing assemblages using relative abundance reveals a combination of species declines and increases that may, or may not, “average out” major individual populations changes (Dornelas et al., 2019). This is clearly consistent with findings from our long‐term study, where significant changes in assemblages revealed through relative abundance were “missed” when examined using presence/absence measures.

### 4.2 Patterns of change

The southwest of Western Australia is currently one of 36 global biodiversity hotspots, regions containing over 1500 species of endemic vascular plants and having lost at least 70% of original habitat due to human activities (Myers et al., 2000), and it is experiencing rapid growth in agricultural, urban, and peri‐urban development across its uniquely evolved landforms and habitats. Habitat modification, fragmentation, and isolation of natural vegetation and biotic communities are a consequence of Perth's expanding footprint (How & Dell, 1993, 2000; Stenhouse, 2004), with concomitant loss among native species. Associated with these anthropogenic impacts is the marked decline in rainfall over the last 50 years that reached 25% below the long‐term mean between 2006 and 2010.

This continuous 35‐year study of reptiles has illustrated minor changes in species richness but marked changes in species relative abundance. Nearly, 75% of known taxa were recorded in any sampling year and assemblages did not change through time analyzing presence/absence data, despite local extinction of three species. However, significant changes were revealed in the Park's assemblages when analyzing relative abundance data. Corroborating this result, four sampling sites representing distinct vegetation communities showed limited assemblage differences analyzing presence/absence data but eight significantly different assemblages using relative abundance data, principally determined by the common taxa present. These contrasts in outcomes from different β‐diversity measures have important ramifications for interpretation of assemblage changes in both space and time as well as conservation and management of faunal populations.

Clearing of natural habitats and fragmentation of others are inimical to the survival of native species that are dependent on the persistence of natural communities and ecosystems for their survival and evolution. Fahrig (2003) stated that habitat loss had large and consistently negative effects on biodiversity, while fragmentation had much weaker effects that were equally likely to be either negative or positive. The local extinction of two snakes and a gecko (probably an agamid also) in Bold Park during the last 35 years supports the documentation of How and Dell (2000) of a decline in both snakes and non‐skink lizard species in urban bushlands over 170 years since European settlement. These local extinctions are further complemented by studies on adjacent offshore islands. Both Rottnest (1700 ha) and Garden (1100 ha) Islands are located less than 30 km from the adjacent mainland and Bold Park, having been isolated for around 6500 years since the last glacial maxima (Lewis et al., 2013). Both islands support far fewer snake and non‐skink lizard species than does the much smaller central Bold Park (~338 ha) and provide an informed trajectory for future local extinctions (extinction debt) on nearby mainland isolates. The loss of up to four species from the Bold Park assemblage over 35 years altered species richness, but the assemblage changes were a response by total species relative abundances to altered rainfall patterns.

Of our four sampling sites, only the Tuart woodland had a significant and constantly divergent assemblage to the others. That was attributable to markedly different edaphic factors supporting the woodland and, in part, the intense fire that burnt the site in December 2000. The fire did not decrease species richness post‐fire, however, 5 years elapsed before the relative abundance returned to pre‐fire levels; a recovery time supported by the pattern of change in the assemblage that also took 5 years to return to a stable structure, although not the same assemblage as recorded pre‐fire. These results contrast markedly with findings from the nearby Kings Park in Perth where smaller burnt sites took just 2 years to return to pre‐fire abundance levels and 3 years for species richness to recover (Davis & Doherty, 2015). Interestingly, temporal changes across assemblages in Bold Park's four sampling sites had significantly similar patterns that were broadly directional, irrespective of habitat type or fire impacts, further implicating changing rainfall pattern as a likely driver of reptile assemblage change, rather than habitat differences or disturbance impacts.

All populations and assemblages experience temporal variation but determining if this is induced by anthropogenic activities or a consequence of underlying natural successional change requires long‐term studies (Ramalho & Hobbs, 2012). While we cannot exclude successional changes in vegetation structure and composition, our study indicates that reptile assemblages in different habitats responded similarly over time, suggesting that external climatic factors are significant drivers of change. This is corroborated by overall reptile relative abundance, which showed a highly significant positive correlation with annual rainfall residuals, specifically with residuals for 4 years preceding a sampling period. This is unlikely to be a spurious relationship as it is supported by known ecological responses among plants and reptile populations in the region's Mediterranean climate.

### 4.3 Demography of change

Our illustration of the positive correlation between annual rainfall residuals and overall reptile relative abundance during the last 28 years is supported by known demographic responses in reptiles across southwestern Australia (Aplin & How, 1993). We confirmed that maximum reptile activity occurs over the warmer months of October to April, peaking during November–December when reproduction occurs in both insectivorous (Davidge, 1980) and saurophagous species (Strahan et al., 1998). Activity ceases, or is markedly reduced, over the cooler, wetter months of late autumn through to early spring when 77% of rainfall occurs. Above‐average rainfall events and concomitant elevated net primary and secondary productivity can trigger increased reproductive effort through increased clutch size or repetitive clutching (How et al., 1986). Survival is probably low among both eggs and juveniles (Kitchener et al., 1988), the latter hatching in the autumnal months (Chapman & Dell, 1985). Maturity varies among species with some species becoming reproductive in their first‐year post‐hatching (Bradshaw, 1971), others only in their second (Davidge, 1980) or third year (How & Shine, 1999) while some geckos reach maturity in their fifth year (Kitchener et al., 1988).

Annual rainfall is the abiotic factor determining soil moisture, plant growth, and ultimately net primary productivity. This, coupled with increasing temperature, leads to a subsequent response in herbivore and detritivore invertebrate abundance during the spring (Harvey et al., 1997; Vidal & Murphy, 2018). Changes in abundance of these invertebrate secondary consumers impact the available dietary resources for most reptile species, the majority being principally arthropod feeders. Resource availability clearly impacts reptile demography through changes in egg production and probably clutch and hatchling size and success.

Within a sampling year, reptile abundance is unlikely to change as a consequence of the rainfall during the year sampling commences (Year 0) as hatchlings (a product of egg production and survival) do not emerge until autumn in the sampling period. However, relative abundance in the following year will alter, dependent on reproductive output and hatchling survival from the previous year. The change in relative abundance will likely be higher if above‐average rainfall occurred the previous year, or lower if it was a drier year. Consequently, reptile relative abundance in a subsequent sampling period is positively correlated with rainfall residual of the previous year (Year −1). This same rationale follows for rainfall residuals occurring for two (Year −2) and likely up to four (Year −4) years prior, noting different maturity schedules for composite species in the assemblage. Reptile dietary resources and demographic parameters (reproduction, hatchling/juvenile survival, and age at maturity) determine relative abundance in subsequent years with these factors influenced by rainfall during years preceding a sampling period, consequently, supporting the documented modeled correlation between annual rainfall residuals and reptile abundance.

## 5 CONCLUSIONS

Understanding temporal change in species composition and assemblages is fundamental to distinguishing change that can be attributed to external factors, such as anthropogenic or climatic factors, from underlying natural change; long‐term studies are essential to this understanding (Butt et al., 2016; Gotelli et al., 2017; Magurran et al., 2010). The rapidly expanding urban area of Perth has profoundly altered fauna in fragmented natural vegetation, facilitating abundant local extinctions over the past 170 years and compounding the extinction debt inherent in remaining natural bushlands. Our results on changes in reptile assemblages concur with the broad generalizations of Dornelas et al. (2014) as we did not detect a dramatic loss in α‐diversity, but an altered community composition (β‐diversity) that changed systematically through time, above expectations. Likewise, the three (possibly four) local extinctions and one colonization that occurred in this study were consistent with Dornelas et al. (2019) who detected accelerating rates of extinction and colonization, with both factors being approximately balanced, thus revealing continuing and increased rates of species turnover.

Our results also indicate a highly significant and positive correlation between rainfall residuals for the years preceding sampling and reptile relative abundance. This climatically induced change in reptile abundance significantly alters assemblages over time. We also show that similar patterns were detected in sampling sites within different vegetation types; a finding consistent with Gotelli et al. (2017) who documented resilience in the temporal trajectories of the assemblages they analyzed.

The combination of the detrimental impacts from long‐term isolation and changed rainfall pattern on species populations and assemblages in southwestern Australia requires consideration when developing conservation and management protocols, particularly if the goal is to maintain biodiversity in a global hotspot that is defined by anthropogenic changes to habitat and which is also experiencing a drying climate. Fragmented urban populations have similarities to island populations with limited opportunities for immigration through a lack of connectivity across the urban matrix such that extinction debts are inherent over time. Under declining rainfall, remnant urban reptile assemblages are even more susceptible to declining numbers and, therefore, local extinctions.

## AUTHOR CONTRIBUTIONS

**Richard Alfred How:** Conceptualization (lead); data curation (lead); formal analysis (lead); funding acquisition (lead); investigation (lead); methodology (lead); project administration (lead); writing – original draft (lead); writing – review and editing (lead). **Jason How:** Data curation (supporting); formal analysis (supporting); methodology (supporting); writing – original draft (supporting); writing – review and editing (supporting). **Mark Cowan:** Formal analysis (supporting); methodology (supporting); writing – original draft (supporting).

## CONFLICT OF INTEREST

None.

## Data Availability

Observation data are available from the corresponding author upon reasonable request or https://doi.org/10.5061/dryad.mgqnk9927.
